# Integrating Cartilage Explant Culture with Simulated Digestion and Hepatic Biotransformation Refines In Vitro Screening of Joint Care Nutraceuticals

**DOI:** 10.3390/mps8040091

**Published:** 2025-08-06

**Authors:** Michelina Crosbie, Kailey Vanderboom, Jamie Souccar-Young, Wendy Pearson

**Affiliations:** Department of Animal Biosciences, University of Guelph, Guelph, ON N1G 2W1, Canada; crosbiem@uoguelph.ca (M.C.); vanderbk@uoguelph.ca (K.V.); jsouccar@uoguelph.ca (J.S.-Y.)

**Keywords:** glucosamine, arthritis, anti-inflammatory, chondroprotective, nutraceuticals

## Abstract

In vitro cartilage explant culture has been used to assess nutraceuticals on cartilage responses to inflammatory stimuli. However, applying extracts of nutraceuticals directly to cartilage explants does not account for effects of digestion and hepatic biotransformation, or selective exclusion of product metabolites from joint fluid by the synovial membrane. The current study produced a simulated biological extract of a common nutraceutical (glucosamine; G**_sim_**) by exposing it to a simulated upper gastrointestinal tract digestion, hepatic biotransformation by liver microsomes, and purification to a molecular weight cut-off of 50 kDa. This extract was then used to condition cartilage explants cultured for 120 h in the presence or absence of an inflammatory stimulus (lipopolysaccharide). Media samples were analyzed for prostaglandin E_2_ (PGE_2_), glycosaminoglycan (GAG), and nitric oxide (NO). Tissue was digested and analyzed for GAG content and stained for viability. Conditioning of explants with G_sim_ significantly reduced media GAG in stimulated and unstimulated explants and reduced nitric oxide production in unstimulated explants. These data provide evidence for the value of glucosamine in protecting cartilage from deterioration following an inflammatory challenge, and the model improves applicability of these in vitro data to the in vivo setting.

## 1. Introduction

Glucosamine-based nutraceutical (GBN) supplements have gained widespread traction in an equine population increasingly plagued by articular diseases such as osteoarthritis [1]. These supplements occupy an important disease-modifying role in managing chronic articular diseases, owing in large part to the absence of a pharmaceutical cure for these conditions, and the relative ease by which consumers can obtain these products. While the availability of GBN supplements for horses is ubiquitous, scientific evidence for their safety and/or efficacy remains less so. This results, in part, from the unbalanced cost–benefit ratio to manufacturers of equine GBN supplements, for which expensive research guarantees neither intellectual property nor its associated market share protection. Research that is conducted on these types of products tends, therefore, to focus on financially frugal in vitro models [2,3,4,5,6,7,8,9,10,11]. These models can offer a cost-effective option to manufacturers motivated to use objective research data to support a knowledge-based marketing strategy and can be useful to characterize direct effects of equine GBN supplements on cartilage tissue. The well-known limitations of all in vitro models notwithstanding, in vitro testing of equine GBN supplements additionally suffers from an inability to account for the effects of nutraceutical digestion, hepatic biotransformation, and passive diffusion of post-hepatic metabolites through the synovial membrane on the response of cartilage tissue to external stimuli.

Cartilage explant is a well-established in vitro model of cartilage homeostasis, which has been used to evaluate cartilage response to inflammatory stimuli under varying conditions. There are more than a dozen studies evaluating glucosamine in cartilage explants, typically under catabolic stress from exogenous stimuli [including lipopolysaccharide (LPS) and interleukin 1β (IL-1), amongst others] [2,3,4,5,6,7,8,9,10,11,12,13,14,15,16,17,18,19,20]. Glucosamine doses used in these experiments range from 10 μg/mL up to 25 mg/mL, with the higher associated with cytotoxicity [3,5] and the lowest with reduced IL-1-induced cartilage degradation [2]. If these concentrations can be compared to that in total body water (approximately 330 L for an average 500 kg horse; [21]), the lowest and highest in vitro doses estimate oral doses of 3.3 g and the supraphysiological dose of 2.72 kg, respectively. The main biological effects of glucosamine in cartilage explant include protection of extracellular matrix structure under pharmaceutical [12] and chemical stress [2,4,5,13,14,16], modulated formation of inflammatory compounds [15,16], and alteration of genes responsible for coordinating biological response to inflammation [6,7,16,17]. However, it is not known to what extent the processes of digestion, absorption, and hepatic biotransformation would influence these outcomes.

We have previously adapted the cartilage explant model to include simulated digestion and hepatic biotransformation steps and have applied this methodology to evaluate the response of cartilage explants to inflammatory stimuli in the presence of polyunsaturated oils [18] and biological active plants [19,20]. The purpose of the current study was to extract glucosamine using simulated digestion and hepatic biotransformation and to quantity effects of this extract on cartilage explant responses to an inflammatory challenge.

## 2. Materials and Methods

All chemical reagents and materials were purchased from Sigma Aldrich (Mississauga, ON, Canada) unless specified otherwise.

### 2.1. Nutraceutical

Powdered glucosamine sulfate K_2_Cl (shellfish source) was provided by Platinum Performance (Buelton, CA, USA). Based on the manufacturers’ recommended daily dose, an initial dose of 8.3 g/day of glucosamine was tested. To assess the dose response of glucosamine, we also tested the product at 3 and 5 times the manufacturer recommended dose (approximately equivalent to doses of 25 and 41.5 g/day, respectively).

### 2.2. Simulated Biological Extract

Based on the initial dose of 8.3 g/day, a simulated biological extract of glucosamine (G_sim_) was created by adding 0.093 g of glucosamine to 17.5 mL of simulated gastric fluid (containing 37 mM NaCl, 0.03 M HCl, and 3.2 mg/mL pepsin) and shaken at 37 °C for 2 h. At 2 h, acidity was neutralized by adding 575 μL of 4.4M NaOH (pH = 6.0) and 18.25 mL of simulated intestinal fluid (containing 20 mg/mL pancreatin, 30 mM K_2_HPO_4_, and 160 mM NaH_2_PO_4_; pH adjusted to 7.4) to the mixture, which was then returned to the shaker at 37 °C for an additional 2 h. The resulting mixture was centrifuged two times at 6000× *g* for 20 min at 4 °C with the supernatant poured off after each spin. The supernatant was allowed to return to room temperature for approximately 30 min then 200 μL of rat liver microsomes (male) were added to reach a final concentration of 0.03 mg/mL [8] followed by NADPH (10 mg/mL in 0.01 M NaOH) [18,19,20]. The resulting mixture was shaken at 37 °C for an additional 30 min then centrifuged at 6000× *g* for 20 min at 4 °C. The supernatant was then returned to room temperature and passed through a 0.22 μm filter and fractioned using a size-exclusion ultrafiltration centrifuge unit (50 kDa; Amicon Ultra). A blank digest (i.e., no product included) was prepared simultaneously using identical methodology. The resulting 50 kDa fractions of G_sim_ were created such that a 10 μL aliquot placed into 1 mL of culture media contained sufficient extract to mimic the initial targeted dose [9]. Final conditioning concentrations in explant wells were 0 (B), 25.1 (T1), 75.3 (T3), and 125.5 μg/mL (T5).

### 2.3. Explant Culture

Explants were prepared and maintained as previously described [18,19,20]. Briefly, articular cartilage from 9 pigs was aseptically harvested from the intercarpal joints using a 4 mm biopsy tool and acclimatized in basal tissue culture media (TCM; comprising DMEM—low glucose supplemented with amino acids, sodium selenite, manganese sulfate, NaHCO_3_, and ascorbic acid) for 72 h in 24-well tissue culture plates (2 explants per well) at 37 °C with 7% CO_2_. Three additional cartilage explants were harvested at the beginning of the experiment, stored in sterile filtered phosphate-buffered saline, and frozen at −20 °C for baseline tissue glycosaminoglycan (GAG) analysis. Media (1000 μL) was removed and refreshed from each well every 24 h. After the first 24 h, G_sim_ was added to fresh TCM every 24 h at doses corresponding to 0, 1, 3, and 5 times the initial dose. After 72 h of culture, half of each of the explant wells were stimulated with lipopolysaccharide (LPS; 10 μg/mL) for the final 48 h. For the final 48 h, media samples were collected prior to LPS stimulation (0 h), and then at 24 and 48 h after LPS stimulation and stored at −20 °C until analysis. At the end of the experiment one explant per well was stained to determine cell viability (see below) and one explant was placed in sterile filtered phosphate-buffered saline and frozen at −20 °C for subsequent analysis of tissue GAG.

### 2.4. Sample Analyses

Tissue culture media samples were analyzed for biomarkers with importance in cartilage inflammation (PGE_2_ and nitric oxide) and structure (GAG release in media and GAG retained in tissue) and assessment of chondrocyte viability (differential live/dead staining). All assay plates were read on a Victor 3 1420 Microplate Reader (Perkin Elmer, Woodbridge, ON, Canada) and concentrations of all biomarkers were determined as follows.

### 2.5. Cell Viability

Viability of cells within cartilage explants was determined using a modified Calcein-AM (C-AM)/Ethidium homodimer-1 (EthD-1) cytotoxicity assay kit (Molecular Probes) modified for use in cartilage explants [18,20]. Calcein-AM and EthD-1 were mixed in sterile distilled water at concentrations of 4 and 8 μM, respectively. Explants were placed one per well into a sterile 96-well microtiter plate and incubated in 200 μL of the C-AM/EthD-1 solution for 40 min at room temperature. The microplate reader was set to scan each well, beginning at the bottom, using 10 horizontal steps at each of 3 vertical displacements set 0.1 mm apart. C-AM and EthD-1 fluorescence in explants were obtained with using excitation/emission filters of 485/530 nm and 530/685 nm, respectively. Viability was determined by the following equation: C-AM/C-AM + EthD-1

### 2.6. Nitric Oxide

Nitrite (NO_2_-), a stable oxidation product of nitric oxide (NO), was analyzed by the Griess reaction [1]. Undiluted TCM samples were added to 96 well plates. Sulfanilamide (0.01 g/mL) and N-(1)-Napthylethylene diamine hydrochloride (1 mg/mL) dissolved in phosphoric acid (0.085 g/L) was added to all wells, and absorbance was read within 5 min at 530 nm. Sample absorbance was compared to a sodium nitrite standard. A best-fit linear standard curve was developed for each plate (R^2^ ≥ 0.99), and these equations were used to calculate nitrite concentrations for samples from each plate.

### 2.7. PGE_2_

Tissue culture media samples were analyzed for PGE_2_ using a commercially available ELISA kit (Arbour Assays; cat #K051-H5; Cedarland Labs, Mississauga, ON, Canada). Plates were read at absorbance of 450 nm. A best-fit 3rd order polynomial standard curve was developed for each plate (R^2^ ≥ 0.99), and these equations were used to calculate PGE_2_ concentrations for samples from each plate.

### 2.8. Media Glycosaminoglycan

Tissue culture media GAG concentration was determined using a 1,9-Dimethyl Methylene Blue (1,9-DMB) spectrophotometric assay [18,19,20]. Samples were added to 96-well plates at 50% dilution and serially diluted 1:2 up to a final dilution of 1:64. Guanidine hydrochloride (275 mg/mL) was added to each well followed immediately by addition of 150 μL DMB reagent. Absorbance was measured at 530 nm. Sample absorbance was compared to that of a bovine chondroitin sulfate standard (Sigma, Oakville, ON, Canada). A best-fit linear standard curve was developed for each plate (R^2^ ≥ 0.99), and these equations were used to calculate GAG concentrations for samples on each plate.

### 2.9. Tissue Glycosaminoglycan

Explants harvested at baseline and after 120 h of culture were digested in papain prior to analysis by 1,9-DMB. Cartilage explants were dried and weighed, and then each disk was cut into 6–8 pieces and placed in microcentrifuge tubes. In each microcentrifuge tube, 600 μL of working digestion solution was added containing 2.6 mg/mL ammonium acetate, 0.38 mg/mL Na_2_EDTA·2H_2_O, 0.31 mg/mL DL-Dithiothreitol, and 40 μg/mL papain (STEMCELL Technologies Canada Inc., Vancouver, BC, Canada). Samples were placed in a shaking water bath for 72 h at 65 °C and checked every 24 h. After 72 h of digestion, samples were frozen at −20 °C until further analysis of tissue GAG.

Tissue GAG concentration was determined using the same methods as media GAG concentrations except tissue GAG concentrations were corrected based on a dilution factor of 30 and as μg/mg of cartilage tissue.

### 2.10. Glycosaminoglycan Retention Index

A Glycosaminoglycan Retention Index (GRI) was calculated for each animal using the following equation:

GRI = Tissue GAG − (∑media GAG for final 48 h of culture)

Values < 1 indicate net loss, and values > 1 indicate net retention.

### 2.11. Statistical Analyses

Data were analyzed using a two-way repeated-measures ANOVA (with respect to time and treatment) to determine the effect of G_sim_ on each outcome measure. A Student *t*-test was used to determine the effect of treatments on cell viability. When a significant *F*-ratio was obtained, the Holm–Sidak post hoc test was used to identify differences between treatments. Data are presented as mean ± SEM unless otherwise indicated. Any treatments stimulated with LPS are designed with + (i.e., B+, T1+, T3+, and T5+). Significance was accepted when *p* < 0.05.

## 3. Results

### 3.1. Cell Viability

**Figure 1:**
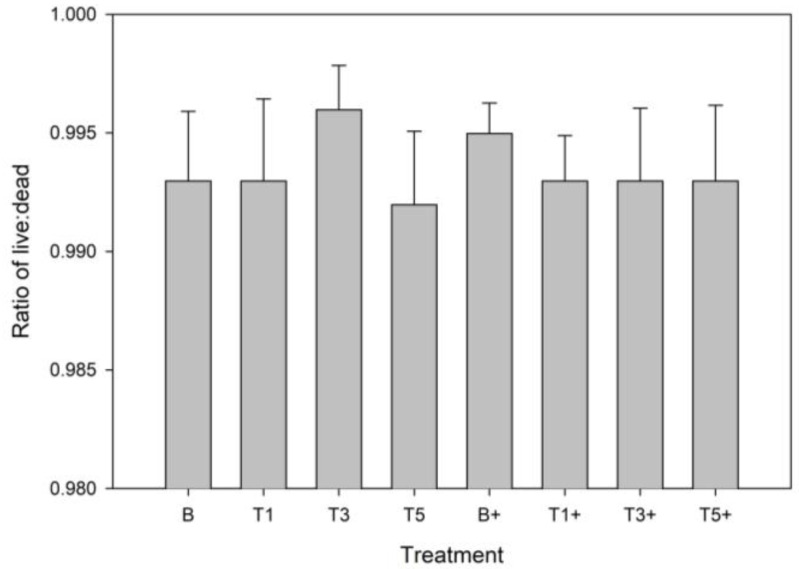
Proportion of live cells within explants conditioned with G_sim_ (T1: 25.1 μg/mL; T3: 75.3 μg/mL; T5: 125.5 μg/mL) and an unconditioned control (B) in the presence (+) or absence of lipopolysaccharide (LPS; 10 μg/mL). Data represent the final 48 h of a 120 h culture duration. LPS-stimulated explants received LPS for the final 48 h.

There was no significant effect of LPS stimulation or G_sim_ on cell viability in explants at any dose (Figure 1).

### 3.2. Nitric Oxide

**Figure 2:**
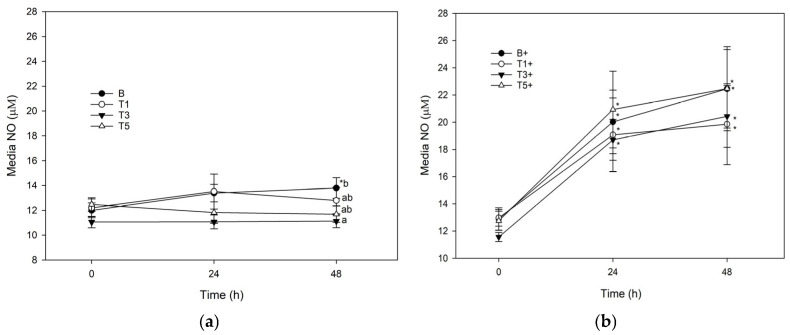
Nitric oxide production from explants conditioned with G_sim_ (T1: 25.1 μg/mL; T3: 75.3 μg/mL; T5: 125.5 μg/mL) and an unconditioned control (B) in the: (**a**) absence of; or (**b**) presence of (+) lipopolysaccharide (LPS; 10 μg/mL). Data represent the final 48 h of a 120 h culture duration. LPS-stimulated explants received LPS for the final 48 h. Lower case letters denote significant difference between groups at a single time point; * denotes significant change from baseline within a single treatment, *p* < 0.05).

Conditioning of unstimulated explants with G_sim_-T3 (75.3 μg/mL) significantly reduced media NO at 48 h (Figure 2a). There was no effect of G_sim_ on NO in stimulated explants (Figure 2b).

### 3.3. PGE_2_

**Figure 3:**
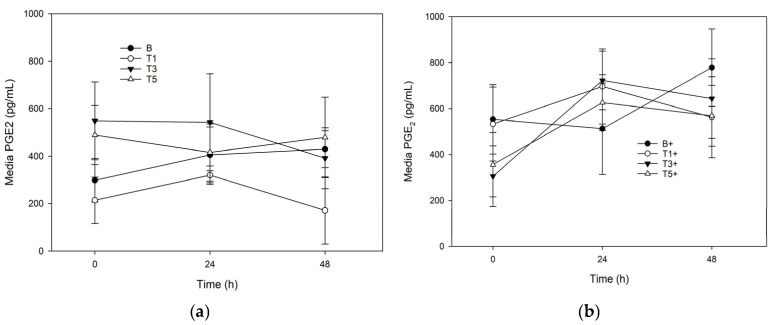
Prostaglandin-E_2_ production by explants conditioned with G_sim_ (T1: 25.1 μg/mL; T3: 75.3 μg/mL; T5: 125.5 μg/mL) and an unconditioned control (B) in the: (**a**) absence of; or (**b**) presence of (+) lipopolysaccharide (LPS; 10 μg/mL). Data represent the final 48 h of a 120 h culture duration. LPS-stimulated explants received LPS for the final 48 h.

Conditioning of explants with G_sim_ had no significant effect on PGE_2_ from unstimulated (Figure 3a) or stimulated (Figure 3b) explants. There were no effects of time or LPS stimulation in any groups.

### 3.4. Media Glycosaminoglycan

**Figure 4:**
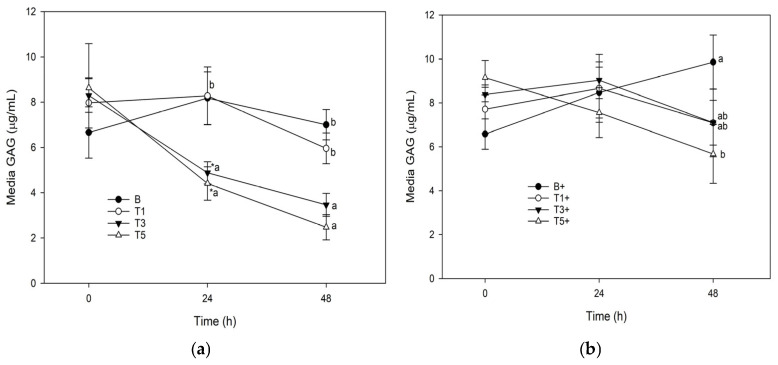
Glycosaminoglycan release from explants conditioned with G_sim_ (T1: 25.1 μg/mL; T3: 75.3 μg/mL; T5: 125.5 μg/mL) and an unconditioned control (B) in the: (**a**) absence of; or (**b**) presence of (+) lipopolysaccharide (LPS; 10 μg/mL). Data represent the final 48 h of a 120 h culture duration. LPS-stimulated explants received LPS for the final 48 h. Lower case letters denote significant difference between groups at a single time point; * denotes significant change from baseline within a single treatment, *p* < 0.05).

Conditioning of unstimulated explants with G_sim_-T3 (75.3 μg/mL) and G_sim_-T5 (125.5 μg/mL) significantly reduced media GAG at 24 h and 48 h (Figure 4a). Conditioning of stimulated explants with G_sim_-T5 (125.5 μg/mL) significantly reduced media GAG at 48 h (Figure 4b).

### 3.5. Tissue Glycosaminoglycan

**Figure 5:**
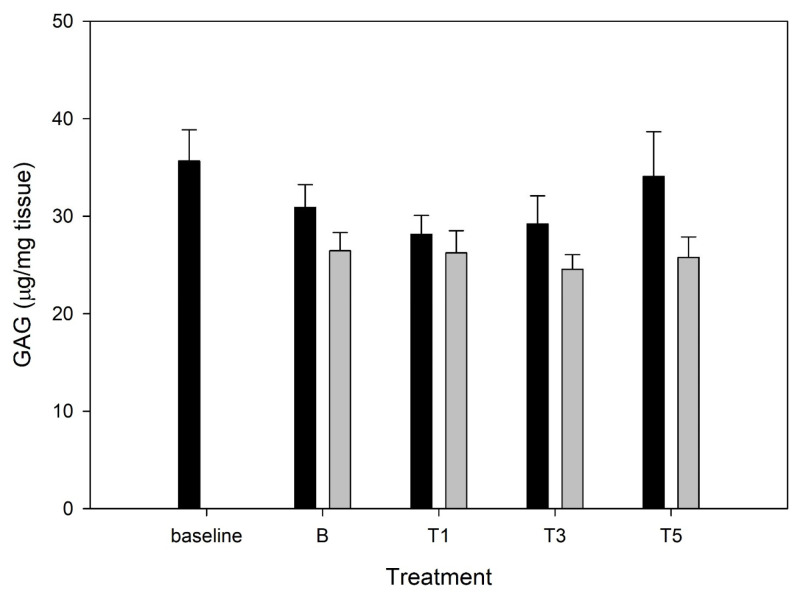
Glycosaminoglycan retention by explants conditioned with G_sim_ (T1: 25.1 μg/mL; T3: 75.3 μg/mL; T5: 125.5 μg/mL) and an unconditioned control (B) in the absence (black bars) or presence (gray bars) of lipopolysaccharide (LPS; 10 μg/mL). Baseline explants were collected prior to 120 h of culture, and all other explants were collected at the end of the 120 h culture duration.

There were no significant effects of time or G_sim_ treatment on tissue GAG (Figure 5). However, it is noteworthy that a dose–response pattern of G_sim_ was observed for increased retention of tissue GAG in unstimulated explants.

### 3.6. Glycosaminoglycan Rention Index

**Figure 6:**
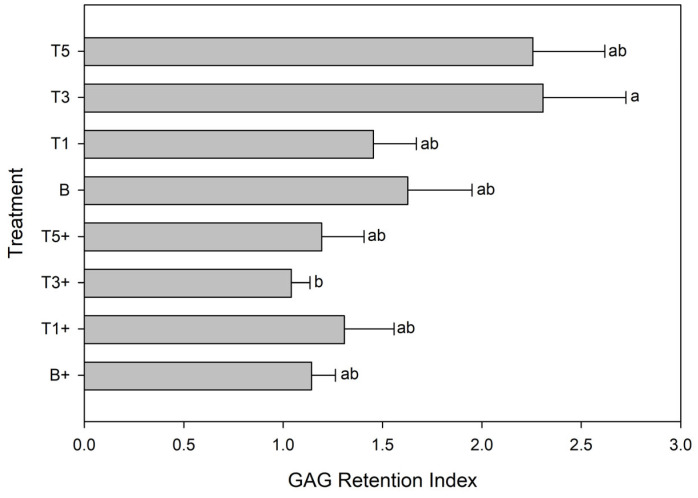
Glycosaminoglycan Retention Index [Tissue GAG / (∑media GAG of final 48 h of culture)] by explants conditioned with G_sim_ (T1: 25.1 μg/mL; T3: 75.3 μg/mL; T5: 125.5 μg/mL) and an unconditioned control (B) in the absence or presence (+) of lipopolysaccharide (LPS; 10 μg/mL). Letters denote significantly different means (*p* < 0.05).

Mean GRI was above 1.0 for all groups, indicating that explants from all animals had a net increase in GAG retention regardless of treatment (Figure 6). The highest and lowest GRI was G_sim_-T3 (2.31 ± 0.42) and G_sim_-T3+ (1.04 ± 0.42), respectively, and the difference between these two groups was significant (*p* = 0.04). There were no other differences in GRI between any other groups.

## 4. Discussion

The purpose of the current study was to extract glucosamine using simulated digestion and hepatic biotransformation, and to quantify the effects of this extract on cartilage explant responses to an inflammatory challenge. The main findings were that conditioning explants with G_sim_-T3 (75.3 μg/mL) and G_sim_-T5 (125.5 μg/mL) resulted in a decrease in media GAG, both in the presence and absence of LPS. Furthermore, the G_sim_-T3 dose resulted in a significant increase in GRI compared with the same dose under LPS stimulation. There was no effect of G_sim_ at any dose on PGE_2_ or NO.

The model described herein represents a significant improvement over conventional explant models, primarily through the incorporation of simulated digestion and hepatic biotransformation steps. This model has been used to evaluate polyunsaturated fatty acids [18] and bioactive plants [19,20], but this is the first time this model has been used to evaluate glucosamine. This is of particular interest because unlike the former test articles, there is an abundance of research on effects of glucosamine in cartilage explant without our modifications, allowing for extrapolations about the role of digestion/hepatic biotransformation on bioactivity of the material. A key difference between the results reported herein and those previously reported for glucosamine [6,7,15,16,17] is the lack of effect of glucosamine on inflammatory biomarkers. We observed no effect of G_sim_ on PGE_2_ or NO, which is in direct contrast with others who report a reduction in IL-1-induced PGE_2_ and/or NO in explants conditioned with 2 mg/mL [2] or 5 μg/mL [16,22] glucosamine. This difference may arise from differing glucosamine doses, or from the use of interleukin 1β as the inflammatory stimulus [2] but may also reflect an effect of digestion/biotransformation/ultrafiltration on bioactivity of glucosamine that was accounted for, at least in part, by our model. However the previously reported effect of glucosamine on glycosaminoglycan dynamics was preserved in this model, and we contribute further evidence for the ability of this supplement to protect cartilage structure in the face of an inflammatory challenge. The inhibitory effect on LPS-induced GAG loss from explants was observed at G_sim_-T3 (75.3) and G_sim_-T5 (125.5 μg/mL), which is approximately equivalent to 25 and 42 g (respectively) for a 500 kg horse (bioavailability notwithstanding). The G_sim_-T3 treatment was also associated with a significant increase in GAG retention in unstimulated explant tissue compared with the same dose in LPS-stimulated explants. The process of explantation exposes cartilage tissue to physical trauma, from which it progressively recovers over the transplantation period [23]. Introduction of LPS into the system interrupts this recovery process and produces a model of injury, while the unstimulated explants continue to recover and produce a model of ‘recovery’. That glucosamine (G_sim_-T3 and G_sim_-T5) markedly reduced GAG loss into media in unstimulated explants, and explants exposed to G_sim_-T3 had the highest tissue GAG, is evidence that this dose of glucosamine reduced loss of GAG from cartilage during recovery from injury. And while the difference between G_sim_-T5+ and G_sim_-T5 did not reach statistical significance, it is plausible that this may have resulted from the higher dose stimulating more LPS-mediated proteoglycan synthesis in stimulated explants, as has been reported by others [13], which may have reduced the difference in GAG retention between stimulated and unstimulated cartilage explants.

Like our observations of GAG and GAG retention, conditioning of explants with G_sim_-T3 also produced a significant decrease in nitric oxide produced by unstimulated explants, but not by LPS-stimulated explants, providing further evidence for the role of glucosamine in cartilage recovery from injury. While nitric oxide at high levels can induce cartilage breakdown during inflammation, low levels can promote cartilage development, demonstrating its dualistic role in cartilage homeostasis [24]. Therefore, interest in therapeutic nitric oxide-donating agents for the treatment of joint diseases like osteoarthritis has increased, but mechanisms of action in the joint remain unclear [25,26]. Glucosamine has been shown to reduce nitric oxide production via inhibition of nitric oxide synthase which likely explains its role in promoting cartilage retention [27]. Others report significant inhibitory effects of glucosamine on nitric oxide release by both unstimulated and stimulated explants [28], but this may have resulted from use of interleukin-1 as a stimulus instead of LPS, and/or from the use of a much higher dose (25 mg/mL) of glucosamine than was used in the current study. It is possible also that the digestion/biotransformation/ultrafiltration processing that glucosamine underwent in the current study also contributed to the differences in responses in the current study compared with earlier studies. Future research should investigate this question through a tandem comparison of G_sim_ and glucosamine extracted without digestion/biotransformation/ultrafiltration in cartilage explants. Alternatively, a comparison of intravenous, intramuscular and/or intra-articular glucosamine effects on nitric oxide in vivo with those obtained by dose-matched oral glucosamine could also provide insight into this question. Intravenous administration of a product containing glucosamine, hyaluronan and chondroitin sulfate resulted in significant worsening of horses in which osteoarthritis had been induced [29], with marginal differences when the chondroitin sulfate was replaced with pentosan sulfate [30]. However, it is unclear to what degree the glucosamine alone contributed to these results. There are in vivo studies which describe a very low bioavailability of glucosamine in horses (approximately 6%) [31,32], but to the authors’ knowledge there are no publications which describe physiological effects of pure glucosamine administered i.v., i.m. or intra-articular in horses, either as a treatment or preventive for cartilage injury. Additionally, the mechanistic action of how digestion and biotransformation impact the bioactivity of glucosamine remains unclear. One study suggests that due to the poor bioavailability of glucosamine in humans, more of it remains available for microbes in the colon which led to an observed shift in the gut microbiome when fed with chondroitin sulfate [33]. As the digestion/biotransformation/ultrafiltration steps used herein did not account for lower gastrointestinal tract digestion, we were unable to determine if this could have impacted our results. Future studies in this area will provide important insight into the ability of our model to account, at least in part, for the dietary nature of glucosamine supplements.

Our study did not generate evidence for a post-hepatic effect of glucosamine on PGE_2_, a key compound in pain signaling, either in the recovery model or the injury model. While this conflicts with in vitro data [34,35,36,37], it is consistent with a large scale in vivo trial in osteoarthritic humans which reports little to no effect of dietary glucosamine on pain [38], poor evidence for any effect on pain in a meta-analysis [39], and a consensus statement by the American College of Rheumatology/Arthritis Foundation which does not support the use of glucosamine to manage pain of arthritis [40]. These types of studies do not exist for horses, but future research in this area may contribute more information to the effects of digestion/biotransformation/ultrafiltration on bioactivity of glucosamine and allow more insight into the in vivo relevance of our data. Limitations of our study are that while the simulated digestion protocol accounts for upper gastrointestinal tract digestion, hepatic biotransformation, and ultrafiltration to account for the applicable particle-size to enter the joint space, this method does not account for lower gastrointestinal tract digestion and microbial metabolism. Additionally, this protocol in conjunction with the cartilage explant model, assumes total and complete dispersion of the nutraceutical into total body water and into the joint space, which may not be the case in an in vivo setting. Regardless, refinement of the in vitro cartilage explant model can provide financially frugal opportunities to explore the safety and efficacy of nutraceuticals in horses prior to their application in vivo.

## 5. Conclusions

Data from this study provide evidence for the usefulness of glucosamine to preserve cartilage structure during recovery from injury, through reduced GAG loss, higher GRI, and lower NO production. The model described herein improves upon conventional cartilage explant methodologies by incorporating simulated digestion, hepatic biotransformation, and ultrafiltration steps and may present an opportunity to better predict the effects of these biological processes on the bioactivity of glucosamine-based nutraceuticals. Additionally, this model provides the opportunity to reduce the financial risk of conducting expensive in vivo trials, thereby increasing our plane of knowledge on the impact these nutraceuticals may have on cartilage health in horses.

## Data Availability

Dataset available on request from the authors.

## Abbreviations

The following abbreviations are used in this manuscript:

C-AM	Calcein-AM
EthD-1	Ethidium homodimer-1
GAG	Glycosaminoglycan
GIT	Gastrointestinal tract
LPS	Lipopolysaccharide
NO	Nitric oxide
PGE_2_	Prostaglandin E_2_
TCM	Tissue culture media

## References

[1] Kawcak C.E., Frisbie D.D., Werpy N.M., Park R.D., McIlwraith C.W. (2008). Effects of exercise vs experimental osteoarthritis on imaging outcomes. Osteoarthr. Cartil..

[2] Bascoul-Colombo C., Garaiova I., Plummer S.F., Harwood J.L., Caterson B., Hughes C.E. (2016). Glucosamine hydrochloride but not chondroitin sulfate prevents cartilage degradation and inflammation induced by interleukin-1α in bovine cartilage explants. Cartilage.

[3] de Mattei M., Pellati A., Pasello M., de Terlizzi F., Massari L., Gemmati D., Caruso A. (2002). High doses of glucosamine-HCl have detrimental effects on bovine articular cartilage explants cultured in vitro. Osteoarthr. Cartil..

[4] Mello D.M., Nielsen B.D., Peters T.L., Caron J.P., Orth M.W. (2004). Comparison of inhibitory effects of glucosamine and mannosamine on bovine articular cartilage degradation in vitro. Am. J. Vet. Res..

[5] Fenton J.I., Chlebek-Brown K.A., Peters T.L., Caron J.P., Orth M.W. (2000). Glucosamine HCl reduces equine articular cartilage degradation in explant culture. Osteoarthr. Cartil..

[6] Ortved K.F., Begum L., Mohammed H.O., Nixon A.J. (2005). Glucosamine hydrochloride and chondroitin sulfate inhibit IL-1-induced gene expression and protein activity in cartilage explants. Osteoarthr. Cartil..

[7] Uitterlinden E.J., Jahr H., Koevoet J.L., Jenniskens Y.M., Bierma-Zeinstra S.M., Degroot J., Verhaar J.A., Weinans H., van Osch G.J. (2006). Glucosamine decreases expression of anabolic and catabolic genes in human osteoarthritic cartilage explants. Osteoarthr. Cartil..

[8] Gouze J.N., Bianchi A., Bécuwe P., Dauça M., Netter P., Magdalou J., Terlain B., Bordji K. (2002). Glucosamine modulates IL-1-induced activation of rat chondrocytes at a receptor level, and by inhibiting the NF-κB pathway. FEBS Lett..

[9] Largo R., Alvarez-Soria M.A., Díez-Ortego I., Calvo E., Sánchez-Pernaute O., Egido J., Herrero-Beaumont G. (2003). Glucosamine inhibits IL-1beta-induced NFkappaB activation in human osteoarthritic chondrocytes. Osteoarthr. Cartil..

[10] Varghese S., Theprungsirikul P., Sahani S., Hwang N., Yarema K.J., Elisseeff J.H. (2007). Glucosamine modulates chondrocyte proliferation, matrix synthesis, and gene expression. Osteoarthr. Cartil..

[11] Piperno M., Reboul P., Hellio Le Graverand M.P., Peschard M.J., Annefeld M., Richard M., Vignon E. (2000). Glucosamine sulfate modulates dysregulated activities of human osteoarthritic chondrocytes in vitro. Osteoarthr. Cartil..

[12] Gulihar A., Shaunak S., Novak P.L., Vinayakam P., Dhinsa B., Taylor G. (2017). Glucosamine reduces the inhibition of proteoglycan metabolism caused by local anaesthetic solution in human articular cartilage: An in vitro study. J. Exp. Orthop..

[13] Phitak T., Pothacharoen P., Kongtawelert P. (2010). Comparison of glucose derivatives effects on cartilage degradation. BMC Musculoskelet. Disord..

[14] McCulloch D.R., Wylie J.D., Longpre J.M., Leduc R., Apte S.S. (2010). 10mM glucosamine prevents activation of proADAMTS5 (aggrecanase-2) in transfected cells by interference with post-translational modification of furin. Osteoarthr. Cartil..

[15] Sumantran V.N., Chandwaskar R., Joshi A.K., Boddul S., Patwardhan B., Chopra A., Wagh U.V. (2008). The relationship between chondroprotective and antiinflammatory effects of Withania somnifera root and glucosamine sulphate on human osteoarthritic cartilage in vitro. Phytother. Res..

[16] Chan P.S., Caron J.P., Orth M.W. (2007). Effects of glucosamine and chondroitin sulfate on bovine cartilage explants under long-term culture conditions. Am. J. Vet. Res..

[17] Chan P.S., Caron J.P., Orth M.W. (2006). Short-term gene expression changes in cartilage explants stimulated with interleukin beta plus glucosamine and chondroitin sulfate. J. Rheumatol..

[18] Garland A., Wierenga C., McCrae P., Pearson W. (2023). Cartilage-Sparing Properties of Equine Omega Complete in an Organ Culture Model of Cartilage Inflammation. J. Equine Vet. Sci..

[19] Pearson W., Kott L.S. (2019). A biological extract of turmeric (*Curcuma longa*) modulates response of cartilage explants to lipopolysaccharide. BMC Complement. Altern. Med..

[20] Pearson W., Fletcher R.S., Kott L.S., Hurtig M.B. (2010). Protection against LPS-induced cartilage inflammation and degradation provided by a biological extract of Mentha spicata. BMC Complement. Altern. Med..

[21] Forro M., Cieslar S., Ecker G.L., Walzak A., Hahn J., Lindinger M.I. (2000). Total Body Water and ECFV Measured Using Bioelectrical Impedance Analysis and Indicator Dilution in Horses. J. Appl. Physiol..

[22] Chan P.S., Caron J.P., Rosa G.J., Orth M.W. (2005). Glucosamine and chondroitin sulfate regulate gene expression and synthesis of nitric oxide and prostaglandin E(2) in articular cartilage explants. Osteoarthr. Cartil..

[23] Pearson W. (2007). In Vitro and In Vivo Methods to Evaluate Putative Anti-Inflammatory Nutraceuticals. Ph.D. Thesis.

[24] Jiang H., Ji P., Shang X., Zhou Y. (2023). Connection between Osteoarthritis and Nitric Oxide: From Pathophysiology to Therapeutic Target. Molecules.

[25] Abramson S.B. (2008). Nitric Oxide in Inflammation and Pain Associated with Osteoarthritis. Arthritis Res. Ther..

[26] Buca B.R., Mititelu-Tartau L., Lupusoru R.V., Popa G.E., Rezus C., Lupusoru C.E. (2016). New Nitric Oxide Donors with Therapeutic Potential. Med.-Surg. J..

[27] Meininger C.J., Kelly K.A., Li H., Haynes T.E., Wu G. (2000). Glucosamine Inhibits Inducible Nitric Oxide Synthesis. Biochem. Biophys. Res. Commun..

[28] Fenton J.I., Chlebek-Brown K.A., Caron J.P., Orth M.W. (2002). Effect of glucosamine on interleukin-1-conditioned articular cartilage. Equine Vet. J. Suppl..

[29] Frisbie D.D., McIlwraith C.W., Kawcak C.E., Werpy N.M. (2016). Efficacy of intravenous administration of hyaluronan, sodium chondroitin sulfate, and N-acetyl-d-glucosamine for prevention or treatment of osteoarthritis in horses. Am. J. Vet. Res..

[30] Koenig T.J., Dart A.J., McIlwraith C.W., Horadagoda N., Bell R.J., Perkins N., Dart C., Krockenberger M., Jeffcott L.B., Little C.B. (2014). Treatment of experimentally induced osteoarthritis in horses using an intravenous combination of sodium pentosan polysulfate, N-acetyl glucosamine, and sodium hyaluronan. Vet. Surg..

[31] Meulyzer M., Vachon P., Beaudry F., Vinardell T., Richard H., Beauchamp G., Laverty S. (2008). Comparison of pharmacokinetics of glucosamine and synovial fluid levels following administration of glucosamine sulphate or glucosamine hydrochloride. Osteoarthr. Cartil..

[32] Laverty S., Sandy J.D., Celeste C., Vachon P., Marier J.F., Plaas A.H. (2005). Synovial fluid levels and serum pharmacokinetics in a large animal model following treatment with oral glucosamine at clinically relevant doses. Arthritis Rheum..

[33] Navarro S.L., Levy L., Curtis K.R., Lampe J.W., Hullar M.A.J. (2019). Modulation of Gut Microbiota by Glucosamine and Chondroitin in a Randomized, Double-Blind Pilot Trial in Humans. Microorganisms.

[34] Kapoor M., Mineau F., Fahmi H., Pelletier J.P., Martel-Pelletier J. (2012). Glucosamine sulfate reduces prostaglandin E(2) production in osteoarthritic chondrocytes through inhibition of microsomal PGE synthase-1. J. Rheumatol..

[35] Frondoza C.G., Heinecke L.F., Grzanna M.W., Au A.Y., Ownby S.L. (2011). Modulation of cytokine-induced prostaglandin E_2_ production in cultures of articular chondrocytes obtained from carpal joints of camels (*Camelus dromedarius*). Am. J. Vet. Res..

[36] Byron C.R., Stewart M.C., Stewart A.A., Pondenis H.C. (2008). Effects of clinically relevant concentrations of glucosamine on equine chondrocytes and synoviocytes in vitro. Am. J. Vet. Res..

[37] Walsh A.J., O’neill C.W., Lotz J.C. (2007). Glucosamine HCl alters production of inflammatory mediators by rat intervertebral disc cells in vitro. Spine J..

[38] Clegg D.O., Reda D.J., Harris C.L., Klein M.A., O’Dell J.R., Hooper M.M., Bradley J.D., Bingham C.O., Weisman M.H., Jackson C.G. (2006). Glucosamine, chondroitin sulfate, and the two in combination for painful knee osteoarthritis. N. Engl. J. Med..

[39] Wandel S., Jüni P., Tendal B., Nüesch E., Villiger P.M., Welton N.J., Reichenbach S., Trelle S. (2010). Effects of glucosamine, chondroitin, or placebo in patients with osteoarthritis of hip or knee: Network meta-analysis. BMJ.

[40] Kolasinski S.L., Neogi T., Hochberg M.C., Oatis C., Guyatt G., Block J., Callahan L., Copenhaver C., Dodge C., Felson D. (2020). 2019 American College of Rheumatology/Arthritis Foundation Guideline for the Management of Osteoarthritis of the Hand, Hip, and Knee. Arthritis Care. Res..

